# “Show me which parasites you carry and I will tell you what you eat”, or how to infer the trophic behavior of hematophagous arthropods feeding on wildlife

**DOI:** 10.1002/ece3.2769

**Published:** 2017-08-17

**Authors:** Boris Makanga, Carlo Costantini, Nil Rahola, Patrick Yangari, Virginie Rougeron, Diego Ayala, Franck Prugnolle, Christophe Paupy

**Affiliations:** ^1^ Laboratoire MIVEGEC UMR 224‐5290 CNRS‐IRD‐UM, IRD Montpellier France; ^2^ Centre International de Recherches Médicales de Franceville (CIRMF) Franceville Gabon; ^3^ Institut de Recherche en Écologie Tropicale (IRET) Libreville Gabon

**Keywords:** *Anopheles*, blood meal, *Plasmodium*, rainforest, trophic behavior, wildlife

## Abstract

Most emerging infectious diseases are zoonoses originating from wildlife among which vector‐borne diseases constitute a major risk for global human health. Understanding the transmission routes of mosquito‐borne pathogens in wildlife crucially depends on recording mosquito blood‐feeding patterns. During an extensive longitudinal survey to study sylvatic anophelines in two wildlife reserves in Gabon, we collected 2,415 mosquitoes of which only 0.3% were blood‐fed. The molecular analysis of the blood meals contained in guts indicated that all the engorged mosquitoes fed on wild ungulates. This direct approach gave only limited insights into the trophic behavior of the captured mosquitoes. Therefore, we developed a complementary indirect approach that exploits the occurrence of natural infections by host‐specific haemosporidian parasites to infer *Anopheles* trophic behavior. This method showed that 74 infected individuals carried parasites of great apes (58%), ungulates (30%), rodents (11%) and bats (1%). Accordingly, on the basis of haemosporidian host specificity, we could infer different feeding patterns. Some mosquito species had a restricted host range (*An. nili* only fed on rodents, whereas *An. carnevalei*,* An. coustani*,* An. obscurus,* and *An. paludis* only fed on wild ungulates). Other species had a wider host range (*An. gabonensis* could feed on rodents and wild ungulates, whereas *An. moucheti* and *An. vinckei* bit rodents, wild ungulates and great apes). *An. marshallii* was the species with the largest host range (rodents, wild ungulates, great apes, and bats). The indirect method substantially increased the information that could be extracted from the sample by providing details about host‐feeding patterns of all the mosquito species collected (both fed and unfed). Molecular sequences of hematophagous arthropods and their parasites will be increasingly available in the future; exploitation of such data with the approach we propose here should provide key insights into the feeding patterns of vectors and the ecology of vector‐borne diseases.

## 1 Introduction

Most of the infectious diseases that have emerged in the last decades are zoonoses originating in wild animals living at low latitudes. Among them, vector‐borne diseases constitute a major risk for human health worldwide (Jones et al., 2008). Understanding the transmission routes of mosquito‐borne pathogens in wildlife crucially depends on documenting the host preferences and blood‐feeding behavior of mosquitoes. This is important for several reasons. First, it allows determining the role and status of different mosquito species in the endemic maintenance or epidemic transmission of vector‐borne pathogens (Apperson et al., 2004; Faraji et al., 2014; Molaei, Armstrong, Graham, Kramer, & Andreadis, 2015). Second, mosquito feeding habits provide information on the ecology of pathogen transmission, leading to more efficient disease and vector control measures for the benefit of animal and human health (Gómez‐Díaz & Figuerola, 2010; Kent, 2009). Finally, knowledge on the spectrum of hosts upon which mosquitoes feed provides important information on the potential risk of interspecies pathogen transfer, and hence on the risk of emergence of novel diseases (Makanga et al., 2016).

Several methods are currently used to study the blood‐feeding habits of mosquitoes: collection of mosquitoes landing on hosts (Takken & Verhulst, 2012), host‐choice experiments (Costantini et al., 1998; Duchemin et al., 2001), or analysis of the blood meal origin in engorged mosquitoes using immunological, molecular or proteomic approaches (Beier et al., 1988; Niare et al., 2016; Townzen, Brower, & Judd, 2008). The first two methods are limited in scope when the number of potential hosts to test is large (e.g. in hot‐spots of high biodiversity), whereas the last method is constrained by the difficulty of collecting adequate and unbiased samples of blood‐fed mosquitoes. Moreover, blood meals can be rapidly digested, which complicates even further the identification of the blood source (Fornadel & Norris, 2008; Muñoz et al., 2012). The majority of blood meal detection methods usually focus on domestic or common host vertebrates, hence normally missing blood meals from more unconventional hosts.

As inferring the trophic behavior of mosquitoes is laden with difficulties, here we propose a complementary indirect approach that exploits information on the parasites carried or transmitted by vector mosquitoes. This strategy relies on the observation that many parasites infect and develop only in specific vertebrate hosts. Therefore, finding one of these host‐specific parasites in a mosquito should provide information about the host species the mosquito has fed on. For instance, haemosporidians of the genus *Plasmodium* are highly specific parasites of a wide range of vertebrates, including lizards, birds, and mammals (Perkins, 2014). Of ~100 haemosporidians that have been described to date, 42, 29, 16, and 9 species parasitize specifically Primates, Chiropterans, Rodents, and Ungulates, respectively (Perkins & Schaer, 2016). Exploitation of information derived from host‐specific parasites was difficult in the past because few nonhuman *Plasmodium* spp. were known before the second half of the twentieth century (Roeder & Anderson, 1990), including species infecting wild mammals such as rodents, bats, ungulates, monkeys, and great apes (Garnham & Heisch, 1953; Thurber et al., 2013). The recent development of molecular screening techniques of host blood and feces, however, has dramatically increased the number of species described, resulting in a plethora of new *Plasmodium* spp., among which those infecting the African great apes (Liu et al., 2010; Prugnolle et al., 2010; Rayner, Liu, Peeters, Sharp, & Hahn, 2011).

Recently, we reported the results of a longitudinal survey carried out in the rainforest of Gabon with the aim of identifying mosquitoes involved in the transmission of great ape malaria parasites (Makanga et al., 2016), as well as of other haemosporidians infecting wildlife (Boundenga et al., 2016). During this survey, more than 2,000 individual anophelines were collected, among which only seven were engorged with blood, and 74 were infected with haemosporidian parasites. Here, we used this dataset to gain insights about mosquito trophic behavior, as a proof of concept of such indirect approach. Specifically, we first analyzed the origin of the blood meals and then inferred the blood‐feeding behavior of unfed mosquitoes from the haemosporidians they were carrying.

## 2 Methods

The sequencing data concerning the haemosporidian parasite screens have already been described in Makanga et al., 2016 and Boundenga et al., 2016; . Therefore, here we provide only some general information about the collection sites and the molecular approach used to obtain such data.

### 2.1 Mosquito collections and identification

*Anopheles* mosquitoes were collected in two wildlife reserves in Gabon (the Lopé National Park and the private game reserve of La Lékédi) that host large natural populations of mammals, including great apes (gorilla, chimpanzee), monkeys (e.g. mandrill, several species of *Cercopithecus* and *Colobus*), ungulates (e.g. red river hog, buffalo, duiker, sitatunga), rodents, and bats. Mosquitoes were sampled using CO_2_‐baited CDC light‐traps positioned in several sites of each reserve corresponding to dense equatorial forest or to forest patches in a forest/savanna mosaic. Traps were operated between 5 p.m. and 7 a.m. from October 2012 to December 2013, totaling 1,620 trap‐nights (see Makanga et al., 2016 for details). Sampled arthropods were killed at −20°C during 1 hr and were then observed under a Leica M80 binocular to (1) identify and isolate *Anopheles* specimens using taxonomic keys (Gillies & Coetzee, 1987), and (2) to detect female mosquitoes engorged with blood. All female mosquitoes were then individually stored at −80°C until they were processed for further molecular analyses.

### 2.2 Blood meal analysis

Host DNA was extracted from the blood contained in female mosquito guts using the Qiagen DNeasy Blood and Tissue kit (Makanga et al., 2016). PCRs to amplify a 415‐bp fragment of the host *cytochrome b* gene (*Cyt‐b*) were carried out in 50 μL reaction volumes containing 5 μL of 10× reaction buffer (Qiagen, Germany), 3 μL of 25 mmol/L MgCl_2_, 1 μL of 10 μmol/L of each primer [*Cyt‐b*‐(f) and *Cyt‐b*‐(r) primers] (Townzen et al., 2008), 1 μL of 10 μmol/L dNTPs, 0.3 μL of DNA polymerase (Qiagen, Germany) and 3 μL of template DNA using a GeneAmp 9700 thermal Cycler (Applied Biosystems, USA) under the following cycling conditions: 95°C for 1 min; 35 cycles at 95°C for 30 s, 52°C for 50 s, 72°C for 1 min, and final extension at 72°C for 5 min. PCR‐amplified products (10 μL) were run on 1.5% agarose gels in 1× TBE buffer for quality control and then sent to Beckman Coulter Genomics (France) for sequencing both strands after purification. Nucleotide sequences were edited and aligned using BioEdit 7.0.9.0 (Hall, 1999) and compared with homologous host sequences contained in GenBank using the basic local alignment search tool (BLAST; http://www.ncbi.nlm.nih.gov) (Altschul et al., 1997). This allowed determining the vertebrate identity of the blood DNA samples. The closest related sequences (all corresponding to wild ruminants with similarity >98%) (Table S1) were used to construct a phylogenetic tree using maximum likelihood (ML) in PhyML v. 3.0 (Guindon et al., 2010), available at the ATGC bioinformatics platform (http://www.atgc-montpellier.fr/). The maximum‐likelihood tree and corresponding bootstrap support values were obtained with PhyML using NNI (nearest neighbor interchange) + SPR (subtree pruning regrafting) branch swapping and 100 bootstrap replicates.

### 2.3 Haemosporidian parasite screening

The presence of haemosporidian parasites in the anopheline samples was detected as described previously (Boundenga et al., 2016; Makanga et al., 2016). Briefly, total DNA was extracted from the mosquito bodies and salivary glands with the Qiagen DNeasy Blood and Tissue kit (Makanga et al., 2016). A nested PCR procedure (Ollomo et al., 2009) was performed using individual DNA templates to detect the parasites and to amplify a 950‐bp portion of their *Cyt‐b* gene. The PCR products of infected mosquitoes were then sequenced as described above. Gene sequences were edited and aligned to published sequences (Table S3) using BioEdit and assigned to known haemosporidian species using maximum likelihood (ML) in PhyML (using ATGC platform). As above, the maximum‐likelihood tree and corresponding bootstrap support values were obtained using NNI (nearest neighbor interchange) + SPR (subtree pruning regrafting) branch swapping and 100 bootstrap replicates. For each individual infection, we deduced the vertebrate host from which the parasite was acquired during a blood meal based on its natural host range.

## 3 Results and Discussion

### 3.1 Blood meal analysis of wild caught *Anopheles*

Among the 2,415 female mosquitoes collected, only seven (0.3%) were engorged with blood, indicating that the baited trapping technique biased the sample toward unfed blood‐seeking mosquitoes. However, in Central African rainforests, it is extremely difficult to collect blood‐fed mosquitoes with alternative sampling tools in a cost‐effective way. The fed specimens belonged to four species: *Anopheles moucheti* (*n *=* *3), *Anopheles marshallii* (*n *=* *2), *Anopheles gabonensis* (*n *=* *1), and *Anopheles obscurus* (*n *=* *1).

**Figure 1:**
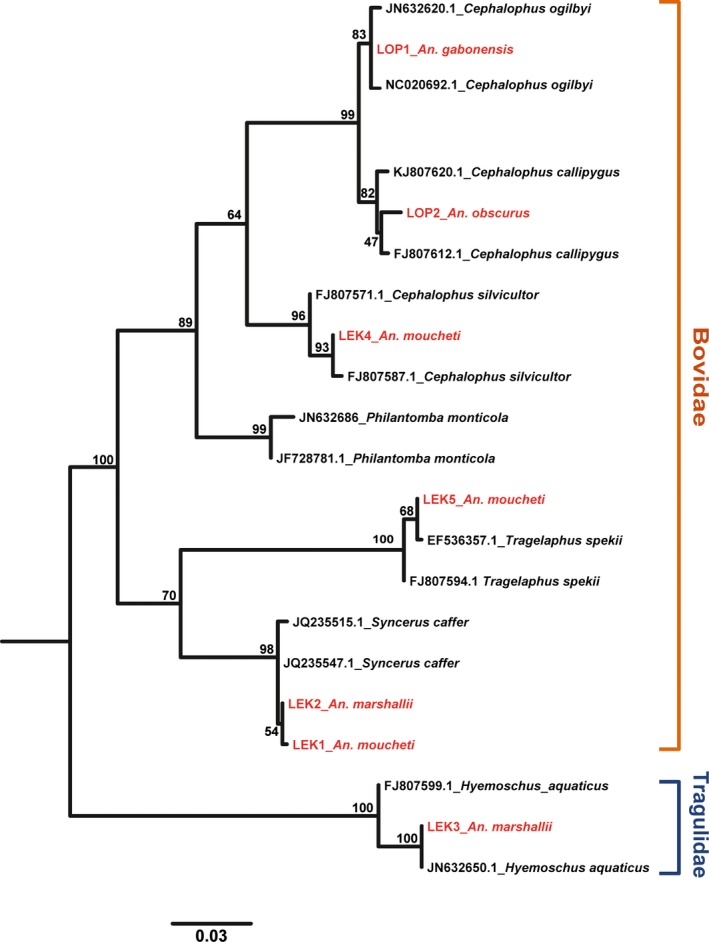
Phylogenetic position of the *Cyt‐b* sequences amplified from the blood meal of field‐collected anopheline mosquitoes (in red: the name is composed from the mosquito species it was recovered and the abbreviation of the collection site, LOP, Lopé National Park; LEK, La Lékédi game reserve) relative to reference sequences for different host species (in black; GenBank sequence number and name of the vertebrate host). Bovidae and Tragulidae refer to the two mammal families the blood originated from. Bootstrap values (100 replicates) are given at each node. Scale bar, 0.06 substitutions per site

The *Cyt‐b* sequence‐based phylogenetic analysis showed that all seven blood meals (in red in Figure 1) originated from forest ungulates belonging to the families of the Bovidae and Tragulidae: *Cephalophus ogilbyi* (Ogilby's duiker, recovered from *An. gabonensis*), *Cephalophus callipygus* (Peters's duiker, recovered from *An. obscurus*), *Cephalophus sylvicultor* (yellow‐backed duiker, recovered from *An. moucheti*), *Tragelaphus spekii* (sitatunga, recovered from *An. moucheti*), *Syncerus caffer* (African buffalo, recovered from *An. moucheti* and *An. marshallii*), and *Hyemoschus aquaticus* (water chevrotain, recovered from *An. marshallii*). These results are informative about the trophic behavior of sylvan *Anopheles* toward wild ruminants in the rainforests of Central Africa and, therefore, about their potential role as vectors of pathogens in wild ungulates. However, the limited number of blood‐fed mosquitoes in our sample, despite the considerable sampling effort, provided an incomplete picture of mosquito–host interactions in this ecological context. To overcome this limitation, we inferred the blood‐feeding behavior of the unfed *Anopheles* by identifying the host‐specific haemosporidian parasites they carried and by relating them to their vertebrate hosts. the footnote for species and rate in bold is: In bold: Infected Anopheline species and corresponding infection rates

### 3.2 Inference of mosquito host‐feeding behavior from haemosporidian infections

**Figure 2:**
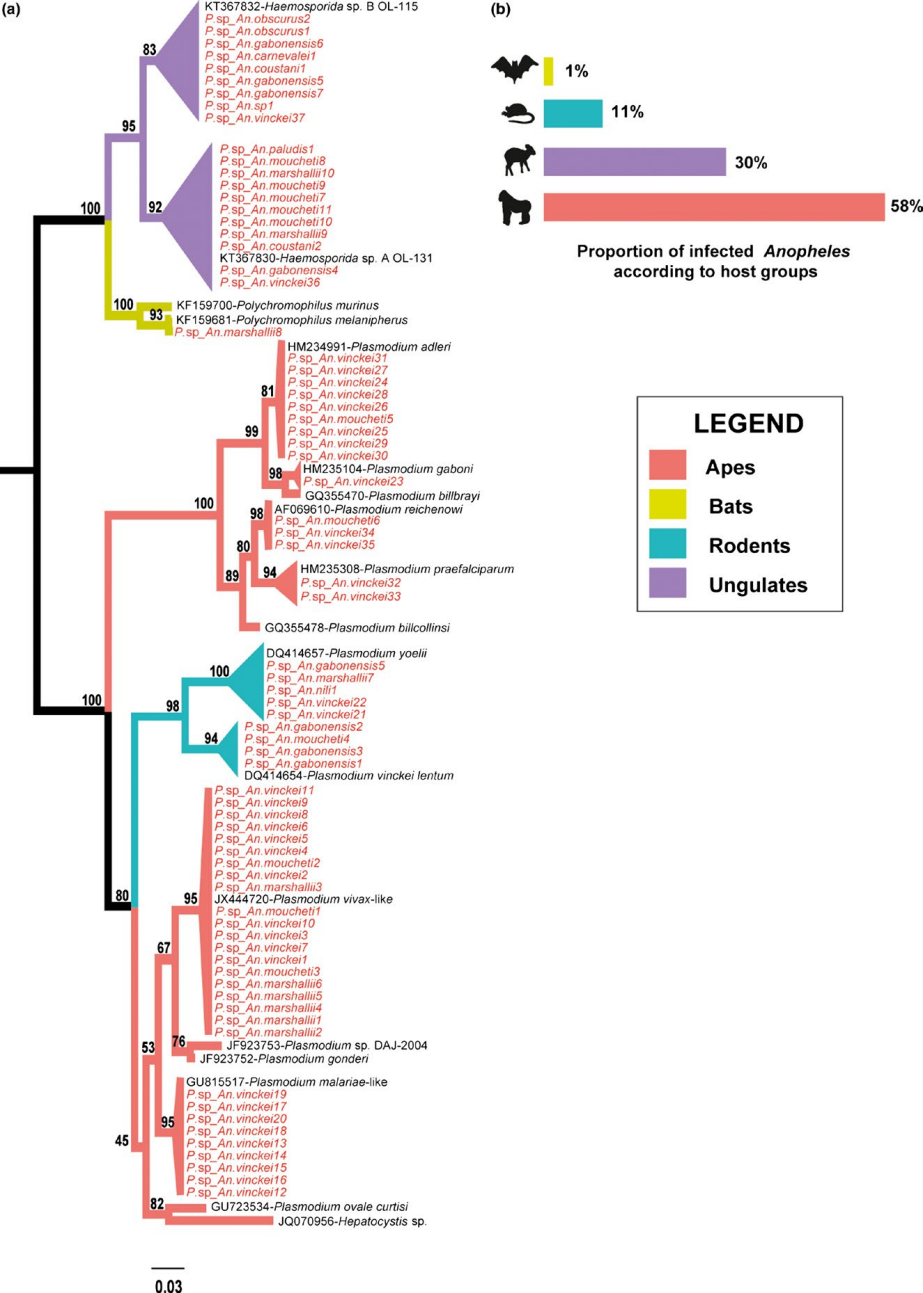
Origin of haemosporidian parasites detected in rainforest anophelines collected in two wildlife reserves in Gabon. (a) Phylogenetic assignation of parasite sequences recovered from field‐collected mosquitoes (in red) to known sequences of Haemosporida (in black) and their natural hosts (see the color coding in the legend). Bootstrap values (100 replicates) are given at each node. Scale bar, 0.05 substitutions per site. (b) Proportion of infected *Anopheles* mosquitoes harboring parasites with vertebrate hosts belonging to one of four different groups of mammals

Toward this aim, the 2,415 female *Anopheles* collected during the survey were screened to identify the presence of haemosporidian parasites. Haemosporida were detected in 74 females (~3% of the sample), and most of them corresponded to parasites known to infect mammalian hosts, in agreement with the mammalophilic feeding behavior typical of the genus *Anopheles* (Bruce‐Chwatt, Garrett‐Jones, & Weitz, 1966). Specifically, they were parasites of African great apes (58%), ungulates (30%), rodents (11%) and bats (1%) (Figure 2a,b, Table S2). Infection rates varied greatly according to the mosquito species and the host group from which the parasites were acquired (Table 1). DNA detection of the parasite infective stages in appropriate tissues (i.e. sporozoites in mosquito salivary glands) was carried out only for a small number of *Anopheles‐*Haemosporida species pairs, thus limiting the scope of inference about the vector role of each mosquito species for these parasites. Nevertheless, the identification of parasite DNA in mosquito tissues necessarily implies that the parasite was acquired from its natural host in the course of a blood meal. The presence of haemosporidians in mosquitoes, therefore, indirectly inform about the trophic behavior of each mosquito species, because most Haemosporida infect a single vertebrate host species or groups of vertebrate species that are taxonomically related. For instance, the rodent *Grammomys poensis* (formerly known as *Thamnonys rutilans*) is the only recognized host for both *Plasmodium yoelii* and *P. vinckei* (Stephens, Culleton, & Lamb, 2012). Hence, mosquitoes harboring these two parasites (in our case *An. marshallii, An. nili,* and *An. vinckei* infected with *P. yoelii*, and *An. gabonensis* and *An. moucheti* infected with *P. vinckei*; see Table S2 and Makanga et al., 2016) must have fed on *G. poensis* or another closely related rodent infected by these parasites. It is not known whether the vertebrate host range of these parasites is much wider, because information about their natural history is quite limited. Nevertheless, it is reasonable to assume that mosquitoes infected by these parasites might have fed on some kind of rodent. Similarly, it can be hypothesized that *Anopheles* infected with *Polychromophilus* spp. (as in the case of *An. marshallii,* see Table S2) should have fed on bats because bats are the only known host of parasites of this genus (Duval et al., 2012; Schaer et al., 2013). The same type of argument can be applied in the case of mosquitoes infected with Haemosporida whose host are wild ungulates or great apes.

**Table 1:** Haemosporidian per cent infection rates according to anopheline species and type of mammalian host

Anopheline species	Proportion of infected mosquitoes by host type					Sample size
	Apes	Bats	Rodents	Ungulates	All hosts	
*An. carnevalei*	0	0	0	**1.8**	**1.8**	56
*An. cinctus*	0	0	0	0	0	2
*An. coustani*	0	0	0	**6.9**	**6.9**	29
*An. demeillioni*	0	0	0	0	0	18
*An. gabonensis*	0	0	**4.3**	**5.8**	**10.1**	69
*An. gambiae s.l*.	0	0	0	0	0	3
*An. implexus*	0	0	0	0	0	31
*An. jebudensis*	0	0	0	0	0	5
*An. maculipalpis*	0	0	0	0	0	1
*An. marshallii*	**0.5**	**0.1**	**0.1**	**0.3**	**0.9**	1,093
*An. moucheti*	**1.0**	0	**0.2**	**1.0**	**2.3**	486
*An. nili*	0	0	**9.1**	0	**9.1**	11
*An. obscurus*	0	0	0	**14.3**	**14.3**	21
*An. paludis*	0	0	0	**1.3**	**1.3**	76
*An. squamosus*	0	0	0	0	0	1
*An. theileri*	0	0	0	0	0	2
*An. tenebrosus*	0	0	0	0	0	1
*An. vinckei*	**7.3**	0	**0.4**	**0.4**	**8.2**	450
*An. *spp	0	0	0	**1.7**	**1.7**	60

Infected Anopheline species and corresponding infection rates

By analyzing the diversity of parasites infecting forest mosquitoes, we could infer different feeding patterns. Some mosquito species had a restricted host range: *An. nili* only fed on rodents, whereas *An. carnevalei*,* An. coustani*,* An. obscurus,* and *An. paludis* only fed on wild ungulates (Table 1, Figure 2a). Other species had a wider host range. For instance, *An. gabonensis* could feed on rodents and wild ungulates, whereas *An. moucheti* and *An. vinckei* targeted rodents, wild ungulates and great apes. *Anopheles marshallii* was the species with the largest host range (rodents, wild ungulates, great apes, and bats). The wider host range of *An. marshallii*,* An. moucheti*, and *An. vinckei* compared with the other species could be partially explained by their greater flight altitude range when host‐seeking: from ground level, where they are likely to encounter rodents and ungulates, to higher levels in the vegetation, where they might encounter primates at night (monkeys and apes), and up to the canopy where bats roost. Conversely, the other six mosquito species probably fly closer to the ground when seeking hosts. As a consequence, host contacts are limited to vertebrates living at this height level, such as rodents and ungulates. Indeed, some mosquitoes are known to have species‐specific vertical distribution patterns when host‐seeking (Haddow, Gillett, & Highton, 1947), or even when dispersing over open ground (Gillies & Wilkes, 1974).

These conclusions should be considered with caution because mosquito host‐feeding patterns depend on many interacting factors, such as inherent host preferences and their modulation by endogenous physiological or exogenous environmental factors, such as the host relative abundance and accessibility (Takken & Verhulst, 2012). Moreover, inference of mosquito trophic patterns from parasite infections suffers from an inherent bias due to vector competence. Indeed, a parasite present in a blood meal cannot be detected after postprandial digestion if it cannot initiate development in the mosquito or until a detectable stage is reached. For example, the absence in our samples of bat parasites in *An. gabonensis* might be explained by their inability to produce oocysts in this mosquito species, rather than to a lack of contact between *An. gabonensis* and bats. In other words, while the presence of a parasite provides robust information about the mosquito feeding behavior, the absence of a parasite only provides ambiguous information. Overall, our results show that the host‐feeding pattern deduced from parasite carriage is congruent with the results obtained by molecular identification of blood meals, while providing additional information that was not otherwise available. For instance, the parasite infection analysis revealed that in the rainforests of Gabon, *An. moucheti* and *An. marshallii* do not only feed on ungulates, but exploit a wider range of vertebrates, something that would have gone undetected based only on the limited information available from the blood meal analysis.

## 4 Conclusions

This study improves our knowledge of the trophic behavior of *Anopheles* mosquitoes living in the pristine rainforest of Central Africa and feeding on wildlife, providing valuable information to understand the transmission pathways of nonhuman Haemosporida. Difficulties with sampling blood‐fed *Anopheles* in this ecological context were at least partially overcome by exploiting information about mosquito‐infecting parasites to infer vector–host interactions. Compared with the conventional analysis of blood‐engorged mosquitoes, this method increased by more than 10‐fold the number of informative mosquito specimens.

The method we propose relies on parasite high specificity in the vertebrate host to determine mosquito blood‐feeding patterns. At the other end of the scale, parasites that are not host‐specific, as it is often the case for enzootic arboviruses (e.g. *Flavivirus* or *Alpha virus*), are uninformative. However, characterization of food networks from mosquito blood‐feeding patterns can serve to predict cross‐species transfer of pathogens showing such lower host specificity. In this context, mosquitoes with opportunistic feeding patterns are of a particular interest because they are able to bridge nonspecific pathogens toward new hosts, including humans. The approach presented here using mosquitoes and haemosporidians as a model can be suitable for other hematophagous arthropods and/or other pathogens infecting wildlife, provided that sufficient information about host specificity is available. Molecular sequences of hematophagous arthropods and their parasites generated from new high‐throughput technologies will be increasingly available in the future; exploitation of such data with the approach we propose here should provide key insights into the feeding patterns of vectors and the ecology of vector‐borne diseases.

## Conflict of Interest

None Declared.
