# Supplementary material for: “Show me which parasites you carry and I will tell you what you eat”, or how to infer the trophic behavior of hematophagous arthropods feeding on wildlife
Source: Ecol Evol. 2017 Aug 17;7(19):7578–84. doi: 10.1002/ece3.2769 (PMC5632637; doi:10.1002/ece3.2769)
Supplement: Supplementary file 1 [file ECE3-7-7578-s001.doc]

**Supplementary Table S1:** GenBank accession numbers of the *Cyt-b* sequences used for the phylogenetic analyses shown in Figure 1. An.: *Anopheles*; LEK: La Lékédi game reserve; LOP: Lopé National Park.

| **Isolates ID** | **Accession number** |
| --- | --- |
| LEK1_*An. moucheti* | KX095987 |
| LEK2_*An. marshallii* | KX095988 |
| LEK3_*An. marshallii* | KX095989 |
| LEK4_*An. moucheti* | KX095990 |
| LEK5_*An. moucheti* | KX095991 |
| LOP1_*An. gabonensis* | KX095992 |
| LOP2_*An. obscurus* | KX095993 |
| *Cephalophus ogilbyi* | JN632620.1 |
| *Cephalophus ogilbyi* | NC020692.1 |
| *Cephalophus callipygus* | FJ807620.1 |
| *Cephalophus callipygus* | FJ807612.1.1 |
| *Cephalophus sylvicultor* | FJ807571.1 |
| *Cephalophus sylvicultor* | FJ807587.1 |
| *Philantomba monticola* | JN632686.1 |
| *Philantomba monticola* | JF728781.1 |
| *Tragelaphus spekii* | EF536357.1 |
| *Tragelaphus spekii* | FJ807594.1 |
| *Syncerus caffer* | JQ235515.1 |
| *Syncerus caffer* | JQ235547.1 |
| *Hyemoschus aquaticus* | FJ807599.1 |
| *Hyemoschus aquaticus* | JN632650.1 |

**Supplementary Table S2:** Details of *Plasmodium* species detected in *Anopheles* carcasses that were used to infer mosquito blood feeding patterns.

| **Infected *Anopheles*** | **Identified parasite** | **GenBank Accession number of *cyt-b* parasite sequences** | **Host categories** |
| --- | --- | --- | --- |
| *An.carnevalei 1* | *Plasmodium sp* | KU318101 | Ungulates |
| *An.coustani 1* | *Plasmodium sp* | KU318031 | Ungulates |
| *An.coustani 2* | *Plasmodium sp* | KU318032 | Ungulates |
| *An.gabonensis 1* | *Plasmodium vinckei lentum* | KU318033 | Rodents |
| *An.gabonensis 2* | *Plasmodium vinckei lentum* | KU318034 | Rodents |
| *An.gabonensis 3* | *Plasmodium vinckei lentum* | KU318035 | Rodents |
| *An.gabonensis 4* | *Plasmodium sp* | KU318102 | Ungulates |
| *An.gabonensis 5* | *Plasmodium sp* | KU318103 | Ungulates |
| *An.gabonensis 6* | *Plasmodium sp* | KU318104 | Ungulates |
| *An.gabonensis 7* | *Plasmodium sp* | KU318036 | Ungulates |
| *An.marshallii 1* | *Plasmodium vivax*-like | KU318038 | African great apes |
| *An.marshallii 2* | *Plasmodium vivax*-like | KU318040 | African great apes |
| *An.marshallii 3* | *Plasmodium vivax*-like | KU318041 | African great apes |
| *An.marshallii 4* | *Plasmodium vivax*-like | KU318042 | African great apes |
| *An.marshallii 5* | *Plasmodium vivax*-like | KU318039 | African great apes |
| *An.marshallii 6* | *Plasmodium vivax*-like | KU318043 | African great apes |
| *An.marshallii 7* | *Plasmodium yoelii* | KU318044 | Rodents |
| *An.marshallii 8* | *Polychromophilus melanipherus* | KU318045 | Bats |
| *An.marshallii 9* | *Plasmodium sp* | KU318046 | Ungulates |
| *An.marshallii 10* | *Plasmodium sp* | KU318047 | Ungulates |
| *An.marshallii 11* | *Plasmodium sp* | KU318048 | Ungulates |
| *An.moucheti 1* | *Plasmodium vivax*-like | KU318049 | African great apes |
| *An.moucheti 2* | *Plasmodium vivax*-like | KU318052 | African great apes |
| *An.moucheti 3* | *Plasmodium vivax*-like | KU318053 | African great apes |
| *An.moucheti 4* | *Plasmodium vinckei lentum* | KU318054 | Rodents |
| *An.moucheti 5* | *Plasmodium adleri (syn P.gorA)* | KU318055 | African great apes |
| *An.moucheti 6* | *Plasmodium reichenowi* | KU318056 | African great apes |
| *An.moucheti 7* | *Plasmodium sp* | KU318057 | Ungulates |
| *An.moucheti 8* | *Plasmodium sp* | KU318058 | Ungulates |
| *An.moucheti 9* | *Plasmodium sp* | KU318059 | Ungulates |
| *An.moucheti 10* | *Plasmodium sp* | KU318050 | Ungulates |
| *An.moucheti 11* | *Plasmodium sp* | KU318051 | Ungulates |
| *An.nili 1* | *Plasmodium yoelii* | KU318060 | Rodents |
| *An.obscurus 1* | *Plasmodium sp* | KU318105 | Ungulates |
| *An.obscurus 2* | *Plasmodium sp* | KU318106 | Ungulates |
| *An.obscurus 3* | *Plasmodium sp* | KU318107 | Ungulates |
| *An.paludis 1* | *Plasmodium sp* | KU318061 | Ungulates |
| *An.sp 1* | *Plasmodium sp* | KU318109 | Ungulates |
| *An.vinckei 1* | *Plasmodium vivax*-like | KU318062 | African great apes |
| *An.vinckei 2* | *Plasmodium vivax*-like | KU318063 | African great apes |
| *An.vinckei 3* | *Plasmodium vivax*-like | KU318069 | African great apes |
| *An.vinckei 4* | *Plasmodium vivax*-like | KU318064 | African great apes |
| *An.vinckei 5* | *Plasmodium vivax*-like | KU318065 | African great apes |
| *An.vinckei 6* | *Plasmodium vivax*-like | KU318066 | African great apes |
| *An.vinckei 7* | *Plasmodium vivax*-like | KU318067 | African great apes |
| *An.vinckei 8* | *Plasmodium vivax*-like | KU318068 | African great apes |
| *An.vinckei 9* | *Plasmodium vivax*-like | KU318070 | African great apes |
| *An.vinckei 10* | *Plasmodium vivax*-like | KU318071 | African great apes |
| *An.vinckei 11* | *Plasmodium vivax*-like | KU318072 | African great apes |
| *An.vinckei 12* | *Plasmodium malariae*-like | KU318075 | African great apes |
| *An.vinckei 13* | *Plasmodium malariae*-like | KU318074 | African great apes |
| *An.vinckei 14* | *Plasmodium malariae*-like | KU318076 | African great apes |
| *An.vinckei 15* | *Plasmodium malariae*-like | KU318077 | African great apes |
| *An.vinckei 16* | *Plasmodium malariae*-like | KU318078 | African great apes |
| *An.vinckei 17* | *Plasmodium malariae*-like | KU318080 | African great apes |
| *An.vinckei 18* | *Plasmodium malariae*-like | KU318073 | African great apes |
| *An.vinckei 19* | *Plasmodium malariae*-like | KU318081 | African great apes |
| *An.vinckei 20* | *Plasmodium malariae*-like | KU318079 | African great apes |
| *An.vinckei 21* | *Plasmodium yoelii* | KU318083 | Rodents |
| *An.vinckei 22* | *Plasmodium yoelii* | KU318084 | Rodents |
| *An.vinckei 23* | *Plasmodium gaboni* | KU318085 | African great apes |
| *An.vinckei 24* | *Plasmodium adleri (syn P.gorA)* | KU318087 | African great apes |
| *An.vinckei 25* | *Plasmodium adleri (syn P.gorA)* | KU318088 | African great apes |
| *An.vinckei 26* | *Plasmodium adleri (syn P.gorA)* | KU318090 | African great apes |
| *An.vinckei 27* | *Plasmodium adleri (syn P.gorA)* | KU318089 | African great apes |
| *An.vinckei 28* | *Plasmodium adleri (syn P.gorA)* | KU318091 | African great apes |
| *An.vinckei 29* | *Plasmodium adleri (syn P.gorA)* | KU318093 | African great apes |
| *An.vinckei 30* | *Plasmodium adleri (syn P.gorA)* | KU318086 | African great apes |
| *An.vinckei 31* | *Plasmodium adleri (syn P.gorA)* | KU318092 | African great apes |
| *An.vinckei 32* | *Plasmodium praefalciparum* | KU318094 | African great apes |
| *An.vinckei 33* | *Plasmodium praefalciparum* | KU318095 | African great apes |
| *An.vinckei 34* | *Plasmodium reichenowi* | KU318097 | African great apes |
| *An.vinckei 35* | *Plasmodium reichenowi* | KU318098 | African great apes |
| *An.vinckei 36* | *Plasmodium sp* | KU318099 | Ungulates |
| *An.vinckei 37* | *Plasmodium sp* | KU318100 | Ungulates |

**Supplementary Table S3: *Cyt-b* sequences of parasites used as references for phylogenetic analyses and their Genbank accession numbers.**

| **Isolate ID** | **Genbank accession numbers** |
| --- | --- |
| *Haemosporida sp.* A OL-131 | KT367830 |
| *Haemosporida sp.* B OL-115 | KT367832 |
| *Hepatocystis sp.* | JQ070956 |
| *Plasmodium adleri* | HM234991 |
| *Plasmodium billbrayi* | GQ355470 |
| *Plasmodium billcollinsi* | GQ355478 |
| *Plasmodium gaboni* | HM235104 |
| *Plasmodium gonderi* | JF923752 |
| *Plasmodium malariae-like* | GU815517 |
| *Plasmodium ovale curtisi* | GU723534 |
| *Plasmodium praefalciparum* | HM235308 |
| *Plasmodium reichenowi* | AF069610 |
| *Plasmodium vinckei lentum* | DQ414654 |
| *Plasmodium vivax-like* (Chimpanzee) | JX444720 |
| *Plasmodium yoelii* | DQ414657 |
| *Plasmodium sp.*DAJ-2004 | JF923753 |
| *Polychromophilus melanipherus* | KF159681 |
| *Polychromophilus murinus* | KF159700 |
